# Window of the World: Sino–African Encounters through Copies and Simulations

**DOI:** 10.1177/00219096211019085

**Published:** 2021-05-24

**Authors:** Lesley Nicole Braun

**Affiliations:** Institute of Social Anthropology, University of Basel, Switzerland

**Keywords:** Politics of representation, Africa–China, performance, tourism, alterity

## Abstract

Located in the manufacturing hub of Shenzhen is Window of the World: a Chinese theme park that features miniature copies of heritage sites from around the world. The individuals living within this constructed simulation are imported from diverse countries. They come to work as performers, animating the different cultural pavilions. As such, the transnationalism made possible by this park provides a window through which we can observe cultural interactivity, as well as the ways in which culture is constructed and re-presented. This article examines some of the processes of cultural encounters through copies of commodified cultural heritage. It also sheds light on the ways in which Kung Fu movies circulating in Africa have inspired the imaginations of young people, revealing cultural feedback loops that provide openings for new contact. Grounded in ethnographic research, the findings here are based on interviews with Kenyan and South African contract workers at this theme park. This article explores young people’s pursuits of new opportunities of identity-formation and self-representation, as well as economic stability and forward mobility.

## Introduction

Munching on fried chicken at an American fast-food chain, we faced the iconic David statue. A Chinese flute soundtrack blared through the outdoor speakers. Next to the David stood a sculpture of Vishnu which stood next to a Chinese terracotta warrior. The Eiffel Tower loomed in the distance. Thato, a young man from South African, looked up from his meal and said: ‘there are many countries represented in this park, but Africa is just one big section with a few Egyptian monuments’. Niko interjected: ‘It doesn’t matter, this is entertainment.’ Thato mused: ‘as a kid from South Africa, I only knew China through Kung Fu movies. You know, Bruce Lee? Isn’t that the same as thinking of Africa as one big pavilion?’ Laughter erupted from the group and everyone began trading their favorite Kung Fu film scenes. The Window of the World theme park is located on 48 hectares (118 acres) in the Chinese city of Shenzhen. Constructed in 1993, it boasts 130 miniature reproductions of famous landmark tourist attractions from around the world, such as Versailles, Mount Rushmore, Persepolis, Angkor Wat and the Egyptian pyramids. In addition, there are what are referred to as Maori and African dwellings, which are performance spaces where interactive shows delight audiences. This for-profit theme park is mainly a rendering of the world outside China intended for domestic Chinese consumption. The park bears an uncanny resemblance to the World Fairs of the 19th century, where crowds were treated to miniature versions of what are now known as United Nations Educational, Scientific and Cultural Organization (UNESCO) heritage sites. What’s more, the fact that the Eiffel Tower itself was constructed when France hosted the World’s Fair in 1889 points to the intersection of temporal and cultural circuits premised on showcasing the nation-state. Since the 1990s, performers from around the world have been living and working year-round within the confines of this Chinese globally themed park. One of the most popular shows is located in the park’s Africa pavilion, featuring a troupe of performers from across the African continent led by a Chinese director who designs the African-style costumes as well as choreographs the African-themed dance. One aim of this article is to explore the processes of cultural encounters exhibited around the world through copies of commodified cultural heritage. Living and breathing inside these ‘copies’, or cultural simulations, are the individuals imported from diverse countries to labor as ethnic performers. Grounded in ethnographic research, the findings here are primarily based on interviews with Kenyan and South African contract workers at this theme park.

While the commodification of local culture has been analyzed with regard to nation-building and statecraft, China is currently hosting a new stage to serve up international culture for tourist consumption. This park offers a window on a Chinese perspective of the world, as choreographed by Chinese entrepreneurs, with state approval. Window of the World reveals Sino–African configurations with regard to the transnational trajectories of individuals seeking new opportunities. These cultural exchanges add new textures to classic postmodern discussions about copies and simulations. Taking as one point of departure Mudimbe’s (1988) landmark oeuvre, *The Invention of Africa*, this article explores systems of representation as they relate to a construction of ‘alterity’. This state of otherness gets complexified in African migrant workers and their performance-labor in the Chinese production of the global stage. This article then suggests that the invention of the ‘other’ occurs through a layered remixing process of replicated and replicable symbols. Though China may not have a history of colonialism in Africa, accusations of neocolonial domination notwithstanding, its history is nevertheless one of conquest and civilizing campaigns, especially with regard to its own domestic populations of ethnic minorities. It seems less revealing to view Shenzhen’s Window of the World as simply a variant iteration of western-style colonial nostalgia, which is simulated through a model of bounded cultures. Rather, a more nuanced reading considers what unique changes occur in this specifically Chinese process of replication. The cultural expressions emergent from the Window of the World theme park are informed by a triangulated relationship between historical context, the global industry of ethnic performance, and the agents who stand to profit.

There is substantial literature about theme parks, ranging from the politics of authenticity (Hoffstaedter 2008; Lukas, 2007; Schlehe et al., 2010; Xie and Wall, 2002) to the circulating essentialized visions of culture (Raz, 2000). Tim Oakes (2006: 168) posits that ethnic performances of the Li people at Chinese theme parks are mimetic replicas of a global tourist model, and reflect a translocal response to global forces. As John L. Comaroff and Jean Comaroff (2009) have shown, locally produced expressions of nation-states are often constructed, packaged and performed in a manner consistent with neoliberal demands for commodified identities. Tourism is premised on both a play with timelessness in the form of nostalgia for a past, and a celebration of the exotic present. Building on what Rosemary Coombe refers to as a ‘postmodern celebration of pastiche and montage-mimetic juxtapositions of alterity’ (1996: 218), this article strives to contribute to these discussions by focusing on the experience of actual individuals entangled in Afro–Chinese encounters. These are individuals who have encountered versions of ‘the other’ through mutual processes of essentialized representation. What is revealed in this process is less a one-way projection and more of a cultural feedback loop. Now, in the time of COVID-19 when travel is restricted, the Window of the World theme park reintroduces the idea of virtual tourism, and expands the discourse of what it means to encounter the ‘other’.

## Methods

Part of a larger study about Sino–African circulations of people and consumer goods, ethnographic research conducted at the Window of the World theme park began just shy of the COVID-19 pandemic. Interviews were carried out with eight performers working for the park. In-person interviews were also done over social media channels such as We Chat and WhatsApp, which became especially important during the pandemic as the park was closed to the public.

The Kenyan and South African interviewees were between the ages of 24 and 29, spoke English and were mostly men save for three women. One performer who had been working in the park for almost six years spoke basic conversational Mandarin as well as some Cantonese. The others had intentions to learn Mandarin, and they cited it as a skill they could use later on in life, especially since, according to them, the Chinese presence is growing in their countries. It should be mentioned that the interviewees were cautious about what they revealed, and closely managed their impressions. They were worried that if they spoke critically about the park, they would offend the park’s management and possibly compromise their employment. As such, pseudonyms were used for all the research participants interviewed for this paper.

## A brief virtual tour of the park

**Figure 1.**
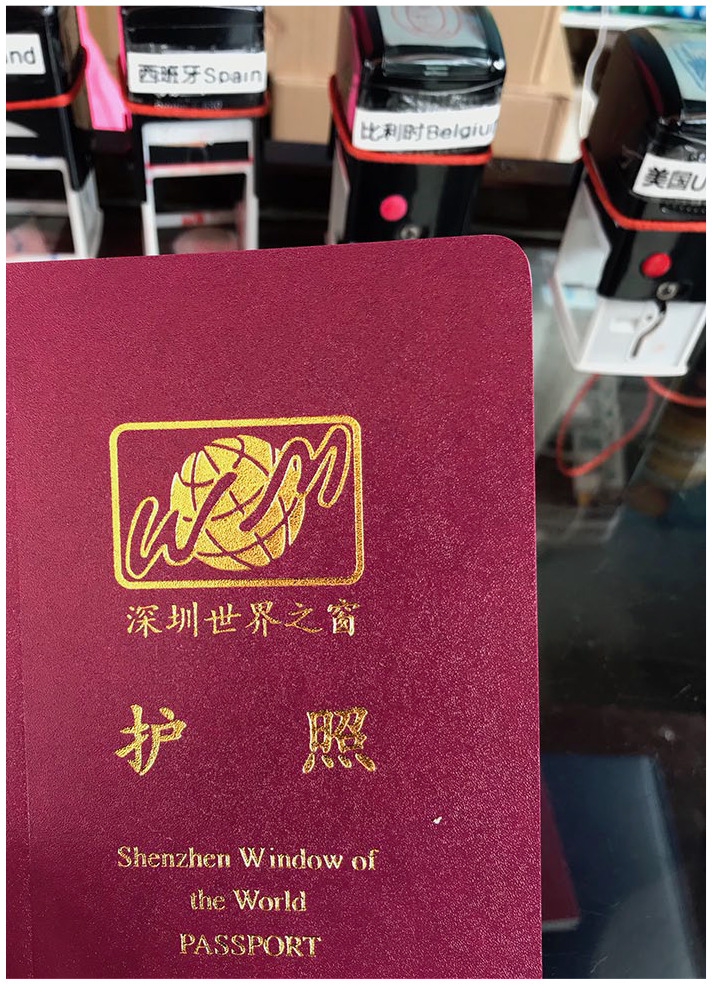
Image of a passport offered for sale at Window of the World. Source: Photo by the author.

Upon entering, it becomes clear that China is at the helm of this experience. Only after passing through the grand ‘China Gate’ will visitors appreciate the world as represented in the rest of the park. For ¥200RMB (price in 2019), the equivalent of US$30, people without the means, opportunity, or desire for real tourism get the chance to see and take photos in front of some of the world’s most famous monuments and landmarks. One can even purchase a passport (Figure 1) and have it stamped at each pavilion – a popular activity among families who roll luggage along to further add to the simulated experience of travel.^1^

The park’s dreamscape of iconic cultural monuments is a veritable studio backdrop, ideal for photos and selfies. One can rent bridal outfits by the church featured in the central square of the European zone for wedding photos, as Whitney Houston’s ‘I will always love you’ plays on a loop to provide the romantic soundtrack to the scene. Costume rentals are available at other pavilions as well, and several attractions even offer iterations of British colonial attire. The center of the park is known as Global Square, with the mini Eiffel Tower marking the large stages for the daily performances. As Corinne Krazt and Ivan Karp (1993: 40) have argued, theme parks offer the opportunity for visitors to ‘imagine, multiply and savor many possible selves’. Indeed, cultural syntax informs the way in which customers interact with the park.

Xudong Zhang and Arif Dirlik offer a succinct description about the particular nature of Chinese postmodernism within a general global capitalist economy:

In the postmodern condition, production and culture mingle with one another through a dialectical third: commodification and consumption. Thus, the encounter with postmodernity is first and foremost a cultural encounter; in other words, the contact with global capitalism is to a great extent the contact with its culture, discourse, ideology, and vice versa. (1997: 12)

The park’s monuments are partly educational in that they all feature short descriptions at each site. The performances, occurring at various locations throughout the day, also aim to be informative about culture and history. At the church, Bible stories are read and interpreted in Chinese. One can be regaled by tales of Greek heroes at the Temple of Delphi and later relax at the Biergarten while listening to German Schlager music. But consistency can just as well give way to pastiche.^2^ By the Taj Mahal, the American hit ‘Old town road’ plays – a song which famously blends the supposed ‘opposing’ genres of country and urban. The pastiche can be temporal as much as spatial. In this regard, perhaps most haunting is the installation of New York City where half the buildings on the Lower East Side are shown in states of collapse, while the Twin Towers, though dilapidated, still stand (as does the Trump tower). There are many kinds of distortions and reductions. Mount Rushmore rises above the Grand Canyon, and yet beyond that horizon lies an area dotted with totem poles devoted to all North American indigenous peoples.

Window of the World is not unlike other parks that offer visitors the experience of commodified sightseeing and globetrotting in miniature. Disney’s Epcot World Showcase, for example, provides the virtual space in which the tourist gaze moves through imagery derived from an ‘elsewhere’ and installed ‘here’ – an elsewhere of space and of time: ‘The deliberate blurring of boundaries between reproduction and fake, fiction and fact, fantasy and history is central to Disney World’s experience and appeal’ (Krazt and Karp, 1993: 32). Likewise, Fredric Jameson (1984) conceptualized a ‘culture of simulacrum’ as a flattening out of surfaces. Add to this Baudrillard’s (1994) claim that what is signified in this world is overwhelmed by the signifier that takes the form of a simulation. In other words, we experience *the real* not just through *the fake*, but through fabricated fakes. The postmodern contention that everything has become a sign liquified of anything ‘real’ maintains its currency here. However, this article argues that the realm of the real is not totally emptied and refilled by the image. Consider the dilapidated quality of the park and what it evokes: given that the park was created in 1993, many of the monuments have fallen into disrepair. But whether it is by design, or because the park will not repair or replace them, is unclear. In this way, the park does not presume to offer a hyper-real experience, but rather the suggestion of a hyper-real experience. In other words, imagination and fantasy are certainly constitutive to shaping the experience, but are in turn shaped by the materiality of the park. The result is a feedback loop between the real and the fictional; we will see a similar loop in our exploration of the work and lives of the African pavilion performers.

A next-level example of the hyper-real experience for this theme park is the inclusion of Disneyland itself in its pantheon of monuments. This meta-statement recalls what Chin-Ee Ong and Ge Jin (2017) note as the ‘double simulation’ occurring in the North Song Dynasty theme park in Kaifeng, China. They, and others, show that idealized historical images from iconic paintings and popular notions of Chinese dynastic history coalesce in the park’s imagery. Likewise, the representations found in the park can also be read as copies of a simulacra model; they are copies of a copy whose relation to the model has become so attenuated that it can no longer be considered to be a copy (Massumi, 1987).

Dubbed as ‘the world’s factory’ by the popular mainstream media, Shenzhen and other cities along the Pearl River Delta mass-produce electronics and other consumer goods for worldwide export. Notably, souvenir trinkets peddled at heritage sites and monuments around the world are also manufactured here, and anyone visiting the pyramids of Giza can buy the Giza keychain made in Shenzhen. China also produces the souvenirs sold at its own simulated pyramids of Giza in Shenzhen, pointing to the movement of iconic monuments as they are disembedded from their original context, copied and thrown into circulation. Umberto Eco (1986), another postmodern scholar, deconstructs the idea of the ‘good copy’, or the absolute fake. Here, hyper-reality occurs when copies of pre-existing models are fabricated to stand in for the real. For artist Hito Steyerl (2009), ‘the poor image’ is a copy in motion:

The poor image is no longer about the real thing – the originary original. Instead, it is about its own real conditions of existence: about swarm circulation, digital dispersion, fractured and flexible temporalities. It is about defiance and appropriation just as it is about conformism and exploitation. In short: It is about reality. (8)

There is some potential in the decreased depth of images compounded with the increased velocity of images. Moving beyond Eurocentric moralizing notions of the profanity of the copy, we observe the ways in which symbols are liquidated of their meaning and then refilled with alterior projections. In the case of Window of the World in Shenzhen, it is the cultural performers who are doing the projecting, as they encounter one another in the space of a world imagined in China.

**Figure 2.**
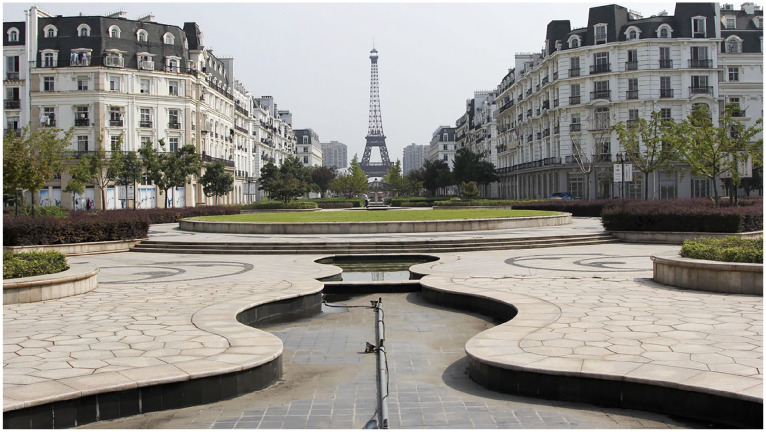
Hangzhou, China urban development replicating Paris. Source: Frank Langfitt/NPR.

Architectural replicas are certainly not uncommon in China. An oft-cited example is found outside the Chinese city of Hangzhou, where reproductions of Haussmann buildings – the Eiffel Tower, the Champs-Élysée – have been constructed for wealthier Chinese residents (Figure 2). Bianca Bosker (2013) refers to China’s penchant for duplicates of western architecture as ‘duplitecture’. She demonstrates the myriad ways in which residents, as well as developers, move through and use these spaces. Gated communities of this variety are popping up all over the country to meet the demands of a rapidly growing middle class. Bosker contends that, for the Chinese government, copies of traditional European styles constitute a means of symbolically conquering the West’s past and present. These buildings, then, become the trophies of such conquest (Bosker, 2013: 2). This problematizes the gesture of superimposing European postmodern theory onto the Chinese context. It falls into the symbolic trap of equating China with the ‘poor image’ or the copy, because it positions Chinese people as merely mimicking hegemonic aesthetics in a manner that postcolonial scholar Homi Bhabha (1984) has critiqued. In this light, understanding the Window of the World monuments through the lens of ‘cheap knockoffs’ associated with China’s infamous counterfeit culture risks cultural essentialism. This is a dubious position given that Europeans have been endlessly copying architecture. Chinese consumption patterns are nudged by the one-party nation-state intent on fostering middle-class lifestyles. Focusing on the Chinese Ethnic Culture Park in Beijing, Ren (2013), a scholar researching the politics of the Chinese middle classes, explores the ways consumerism, tastes and aspirations move between theme parks and gated communities. As China has experienced a series of historic closures and openings, theme parks provide a new kind of opening, one through which people can get a sanitized glimpse of other cultures, without the contaminating effects of having to directly engage with them: ‘They can consume foreign settings without being confronted with foreign people; they can appropriate cultures and history without being faced with political, social, or ecological problems’ (Schlehe et al., 2010: 86). Further, keeping with China’s centralized planning and system of political rule, theme parks like Window of the World allow for a selectively scripted version of the world, curated by the Chinese state.

### ‘People pay to see our skin’: performing Africa

**Figures 3 and 4.**
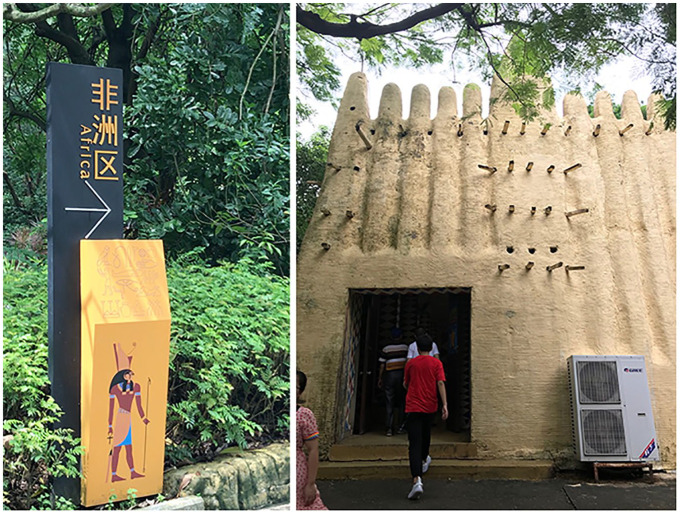
Entrance to the Africa pavilion (left); doorway into the indoor performance hall (right). Source: Photos by the author.

Around the corner from the Egyptian pyramids lies a pavilion dedicated to Africa. It is demarcated by a large structure vaguely resembling the architecture in Timbuktu. The main building is framed by an outdoor performance space surrounded by grass huts where visitors can buy wooden masks and other souvenirs. The pavilion is distinctly rural, giving visitors the impression of being in ‘the bush’. A sign informs visitors that performances are held four times a day, in what is referred to as the ‘African dwelling-house’ (Figures 3 and 4).

A vigorous drumbeat alerts the audience that a show is about to begin inside the air-conditioned amphitheater. Three young African women take the stage, sporting colorful skirts, armbands and beaded headpieces (later they said they were from South Africa). They are accompanied by a Djembe drummer (also from South Africa) who animates the show alongside a male host speaking in Mandarin and Cantonese. This host, a self-identified member of the ethnic Wa Chinese minority, is dressed in a similar costume and is bare-chested. The women dancers punctuate the music with high-pitched shouts and whistles. Once the dance segment ends, an audience member is invited up onto the stage to try playing the drum. The drummer performs a simple tempo for the participant to mimic. It is clearly more difficult than it looks, and the participant struggles to get the rhythm right. Earnestly trying, these participants clumsily beat the drum, amusing the crowd with their rhythmic ineptitude. This interactive portion of the show allows the performers to directly engage the crowd, imbuing the spectacle with an air of playfulness which helps to circumvent the established rigid dynamic between performers and the audience.

Amahle is a 24-year-old trained dancer from South Africa. She used to work for folkloric dance groups across South Africa before being scouted by a white South African agent connected with international theme parks. In fact, most of the performers working at the Africa pavilion have been recruited by brokers who work in ‘heritage performance’ in their respective countries, pointing to long histories of connection and dislocation, linked to colonial enterprises. After about a year of working at Window of the World, she was making plans to return home permanently in the following months. Thato, also a South African, has been working at the park the longest, over six years. He is a self-taught dancer, but he only learned Djembe drumming from another theme park performer while living in China. Thato and Amahle work six days a week, though sometimes on their day off the management still tells them to perform. Thato also frequently performs a solo show on the global stage in the center of the park. For this show, he is presented in an elaborate costume meant to represent an African king. He views these moments as opportunities to showcase his own dance skill to a larger audience. There are other incentives to take on this extra work: these global stage gigs are reserved for senior performers who are especially talented, and, as such, they pay more. This extra remuneration come with other kinds of sacrifices, as Thato explained:

It is difficult to get time off to travel back to South Africa. When I request a leave of absence, especially during the high season, the park convinces me to stay by offering me a salary increase. This money makes it hard to leave. I haven’t visited my home in two years. But then again, I am earning money to send back to my family.

Amahle chimed in, adding that while the salary is good, life can be hard, especially since the performers must live in dormitories on the park’s grounds. She said:

There isn’t much separation from our work because we are always here. The park is very strict. When a performer breaks a rule, they get deductions from their salary. Here, you have to be disciplined if you want to get ahead. We cannot perform outside of the park, even if we are offered opportunities, such as to perform at a party or at a restaurant.

Amhale was well aware of her temporary migrant status, as well as the limitations of working for a Chinese corporation that consistently monitors her whereabouts. She also expressed a desire to explore China’s interior, though she felt that being African would draw considerable attention and make traveling more difficult.

Thato offered his impressions of the Africa pavilion’s host, stressing that he mostly kept to himself. The host not only worked at the Africa pavilion, but also hosted shows in the Maori dwellings. ‘[O]ur host is part of the Wa ethnic group in China’, Thato explained, ‘which is maybe why he has a dark complexion, but sometimes he wears makeup to appear darker in the shows.’ The Wa people in China and Myanmar have long been utilized to serve up the cultural spectacle of minority nationalities (Fiskesjö, 2015). In fact, prior to the importation of African performers, dark-skinned ethnic minority Chinese people were recruited to Window of the World to perform as ‘Africans’, as well as Maoris and American Indians. Here the dark complexions of ethnic minorities have been utilized in portrayals of ‘otherness’ showcased in the live spectacles at Chinese theme parks. Considered ‘wild’ and ‘primitive’, the state instrumentalized this ethnic group to circulate images of themselves and further exoticize their own culture. As Comaroff and Comaroff (2009) have argued in *Ethnicity Inc.*, the intertwined relationship of ethnicity and commerce reveals the ways in which people can economically benefit by taking charge of their own cultural scripts:

Those who seek to brand their otherness, to profit from what makes them different, find themselves having to do so in the universally recognizable terms in which difference is represented, merchandized, rendered negotiable by means of the abstract instruments of the market: money, the commodity, commensuration, the calculus of supply and demand, price banding. (24).

This intentional commodification of exoticized imagery can be read as an expression of self-reflexivity on the part of the Wa people, in that they have some level of say in their own representations. This sentiment was also expressed in interviews with the African performers working at Window of the World who felt a sense of pride in performing the role of virile African kings, queens and warriors. Magnus Fiskesjö argues that, in the face of Wa cultural destruction, as part of the Chinese conquest and annexation of their land, the cultural performances in the Folk Cultural Villages theme park, though deeply commodified, are felt by some young Wa people to be an alternative to total annihilation. For the African performers at Window of the World, willing participation in the commodification of culture is not so much in the service of cultural preservation as it is a means to earn a livelihood abroad, before returning home. Further, in contrast to the Wa performances elsewhere, at the Africa pavilion, commodified imagery is directed by a Chinese art director who dictates the aesthetics of the show. Dance choreographies are also designed and taught to performers by a Chinese man accompanied by a translator.

The newest performers were all young men from Kenya, most of whom spoke limited English. Thato was in charge of orienting this new group. One newly arrived performer explained:

We came to China because we were chosen by a Chinese agent who came to see us perform one evening in Nairobi. We all had interviews with this agent and his team, and in the end we were presented with a contract. Our contract is for one year, but we can renew it annually, if we wish to stay on. I see this as a temporary thing.

Here, the global assumes the form of the Chinese nation-state while the local is represented as the village, and, more specifically, the ethnic village. According to Oakes (2006), out of the 93 theme parks that were cataloged in China in the 1990s, 34 were based on ‘ethnic folk culture’ (179). What’s more, only a stone’s throw from Window of the World is a sister theme park called Folk Cultures Village, also known as Splendid China. While ethnicity may be degraded through its promotion outside temporal and spatial contexts, other scholars point to tourists as another active source of influence. They argue that tourists are not entirely passive, but rather understand (on some level) that they are collectively consuming ‘fakelore’ (Feifer, 1985). At the Splendid China park, ethnic Chinese groups participate, albeit in a coerced manner, in the modern project of urban development. This includes the legitimacy of Chinese citizenship (or at least its promise), as well as the commodification of their culture and knowledge into performances for emergent global tourism. While not entirely analogous, African performers are also signaling their awareness of global tourist models, which shape local economies in their respective countries. The additional transnationalism gets layered on top of the existent Chinese translocal dynamics, and this produces new encounters with the other. This is especially salient against backdrops of racial discrimination. In one group interview at Window of the World, the newly arrived Kenyan dancers mused about some of the imagery employed in the park’s shows. One dancer said: ‘[T]here are so many African cities with skyscrapers. These are not depicted here. It gives the impression that we are still all living in the bush.’ This is reminiscent of what Thato said in another interview:

When I perform, people do not know that the Djembe drum I play on stage is from West Africa, or that the costumes made of fur are from South Africa. They don’t care, they only want to see that we’re black and performing. They want to see that we are African, and not Asians in makeup. I realized this when it was very cold one weekend, and our troupe had to perform in the evening in the freezing cold. All of the other performers, like those from Ukraine and Argentina, were given thin skins to wear under their costumes, but we were not allowed any undergarment because it would mask our skin color. People pay to see our skin.

For Thato, the ‘authenticity’ on display at the park is premised less on the aesthetics of the performances than it is on the shade of his skin which the management demands that he lays bare. Thato’s uncomfortable realization was further reinforced in the host’s use of dark makeup, to look more African whilst performing next to the other dancers.

**Figure 5.**
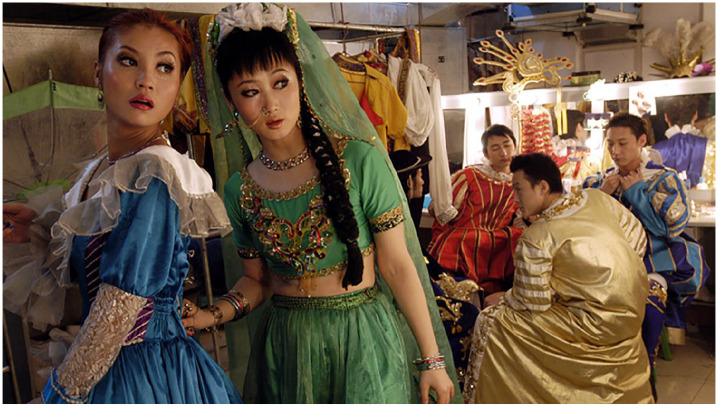
Image still from the film by Zhangke Jia (2004) entitled *The World*.

Performers at Chinese theme parks were the subject of Zhangke Jia’s (2004) film, *The World*. The title points to the Shakespearean notion that ‘all the world’s a stage’, upon which we are all cultural actors in Goffman’s (1956) sense in that one man in his time plays many parts. The set costumes of the mainly Chinese and Russian performers display the exotic quality of not only different cultures represented in the park, but also of the distant past (Figure 5). Though characters are confined to performing in their respective pavilions, Zhangke Jia’s scenes of what goes on backstage present a different image. Here there is movement; at times, even chaos. This theme park can also be considered a ‘life habitat’ as Erik Bordeleau (2010: 168) has described in his analysis of Jia’s film. Bordeleau argues that these settings present an opportunity to think through some of the global processes of representation. The film presents the theme park as a unified and claustrophobic whole – a kind of snow globe in which characters are held almost captive to their own cultural expressions. The boundedness of cultures and of performers to their designated pavilions also points to a longer history of cultural spectacle, most evident in the World Fairs of the 19th century.

**Figure 6.**
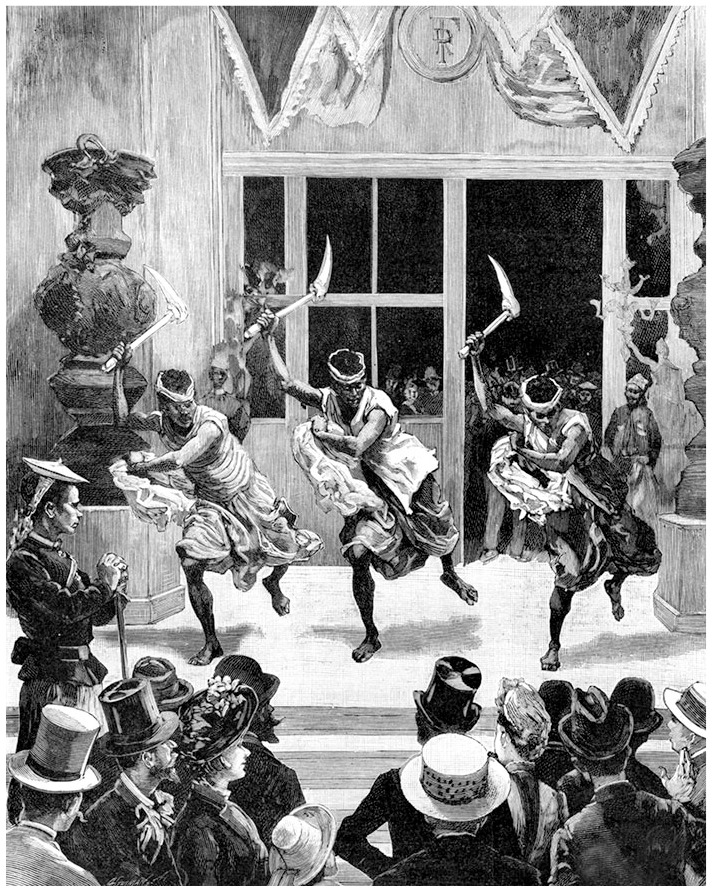
Engraving of a Kanak dance on the Esplanade des Invalides during the 1889 Universal Exposition in Paris. Source: Courtesy of Tallandier/Bridgeman Images.

At the turn of the century, large expositions were held periodically in many major world cities. The intention was to educate and impress audiences, while host countries got to indulge in national hubris. One main aspect of these fairs was to immerse international crowds into a fleeting experience of the entire world in miniature. In addition to the local nationalism on display, these fairs also engendered Eurocentrism. This was simultaneously being reinforced through imperialist projects in faraway ‘exotic’ places. As such, these fairs were concomitant with imperial domination (Figure 6). With new images and conceptions of alterity emerging from European colonial exploration, the European public was thrown into a highly mediated relationship with the exotic other. Exhibits like London’s *Colonial and Indian Exhibition* held in 1886 is one example of this. Even more stark were the African ‘native villages’ and living dioramas (later referred to as human zoos) which were integral to the spectacle of these exhibits intended to stimulate a sense of fear, wonder, and desire for the ‘primitive other’. Audiences, kept at a safe distance, gazed at humans who were forced to perform a version of their way of life in a contained environment which left the European spectator ‘uncontaminated’. These ‘zoos’ represented occasions to publicly other the human body.

Overlapping in various ways, the World Fairs and the contemporary theme park both present a historical recursion that is reminiscent of Walter Benjamin’s (2007) analysis of the emergent dialectic between the original and the copy. In the theme park iteration, performers earn their living by actively performing their own cultures to suit the market-generated fantasies of the audience. The sense of wonder cultivated in contemporary Chinese theme parks is largely found in the otherness exhibited by performers from all over the African continent – not their own specific ethnicities, but rather an all-in-one ethno-image packaged for sale through a process of amalgamation. The performers at the Africa pavilion understand their spectacle to be one that reflects a Chinese-imagined Africa. Tina, one of the performing women, explained: ‘we’re performing in the style of the Lion King, or even Black Panther, and the art director takes what he thinks Chinese people imagine to be Africa, maybe from Safaris and such’.

African entrepreneurs who come to China on business trips comment frequently on the striking ways in which they are othered in public (Huynh and Jung Park, 2019). Accounts of Chinese pedestrians touching their hair and skin, or even changing seats to avoid sitting next to Africans on buses and metros, are only a few examples. Many of these African migrants know it is because they are seen as a novelty among people who do not frequently interact with foreigners. Window of the World therefore provides people with an opportunity to experience a sanitized performance by Africans themselves. Perhaps the most uncomfortable similarity between the World Fairs and this theme park is the fact that performers are lodged within the park itself, rendering it a kind of life habitat in which the confined people are reduced to living objects of the gaze. This contained environment is also reflected in those real-estate developments, particularly in the gated residential community. Here again, people safely dwell in a protected space, away from the contaminating other, and, in this case, the lower economic classes who are kept at bay. Culture is thereby kept in its respective place, while all that suggests an ‘elsewhere’ is integrated into quotidian life and reflected in the built environment through replicas of European architecture. This relates to a longer history of containment, as exemplified in the southern province of Canton which had been historically walled off to keep its population isolated and protected from international merchants and invaders. Here, the closed-in quality that capitalism engenders has the potential to wall off, and to wall in, parts of the world. Against the globalist narratives of the 1990s, culminating in China’s admittance into the World Trade Organization (WTO) in 2001, theme parks reaffirm the narrative of ‘It’s a small world, after all’, as well as simplify the world’s diversity through a process of categorization and compartmentalization. China is not alone in containing its own version of the world. Even within the park, it is not just the ground rules which apply; each cultural group tends to stick to its respective demarcated villages.

## ‘Be like water’: postcolonial encounters with Kungfu

For many individuals, mass media and pop culture (like movies) are a primary point of encounter which shape perceptions of the ‘other’, as opposed to the exclusive world of international business and global political economy. This section turns to Afro–Sino relations motivated by pop culture, and, specifically, to the Kung Fu genre. Such popular imagery has been drawn into circles of representation in both Africa and China. The memetic pull of these movies has given rise to simulated expressions by Africans. Kung Fu consequently draws them into a feedback loop, from which they go on to perform their own versions of Kung Fu choreography for Chinese audiences. The recursion of form is not geometrical; rather, it is entangled in a web of influences that are refracted from many sources, at different angles and within various spatialities. The result is another iteration of the *suggestion of the hyper-real*, where the copy does not necessarily replace the original, but the two mutually alter each other in an ongoing dialectic.

Recent understanding of Global South triangulations explores Afro–Sino relationships that disrupt old binaries, such as the colonial systems of satellite and metropole, as well as race and racism more broadly (Castillo, 2020; Huynh and Jung Park, 2019; Lan, 2017; Monson, 2013). Disruptions in colonial hierarchies of language apparent in postcolonial literature point to new perspectives on overlapping temporalities within a global capitalist framework (Yoon, 2020). Likewise, performers at Window of the World reveal the complexities of cultural encounters and representation through triangulated arrangements involving popular visual culture such as films. Since the Cold War, China has been making its presence felt throughout African mediascapes (Banda, 2009), including infrastructure, news media and entertainment. Films from the 1970s featuring actors like Bruce Lee, Jackie Chan, and later Jet Li, have been captivating crowds all over the African continent with their cinematic martial arts and acrobatics. Reminiscing about his own cinematic experiences, one performer at Window of the World offered this: ‘for as little as 5 Kenyan shillings we got to see a Kung Fu movie dubbed in Swahili. But we didn’t really care about the language, we loved to see the action.’ In fact, most of the performers interviewed spoke of their early impressions of China as being formed from these movies they saw as children. As one interviewee said:

I was inspired to become an acrobat after I saw Bruce Lee movies in Nairobi. I mimicked what I saw with my friends, and from then on tried to improve my skills. I later joined a dojo. Eventually I formed a group with other dancers and acrobats to perform at folklore festivals in Kenya. That’s how I got scouted to work in China.

Rather than viewing films as merely an expression of soft power or media imperialism, the circulation of media engenders these novel self-making practices. For young men, martial arts especially resonate with local and cultural images of masculinity. Charles Ambler is one scholar who studied video distribution in Africa and how it intersects with global media capitalism and leisure:

It is notable that young people in Kenya or Nigeria (or the United States, Europe, and Latin America) can communicate through an allusionary vocabulary drawn from Bruce Lee films, just as to a lesser extent their fathers or grandfathers did through the cowboy vernacular drawn from American Westerns. (2002: 122)

Echoing this, one performer said:

The heroes I used to watch in Kung Fu movies are so strong, and they fly across the screen which was really impressive and inspired me so much. This is what I wanted to replicate with my martial arts training. I also liked the way martial arts and dance can go together.

**Figure 7.**
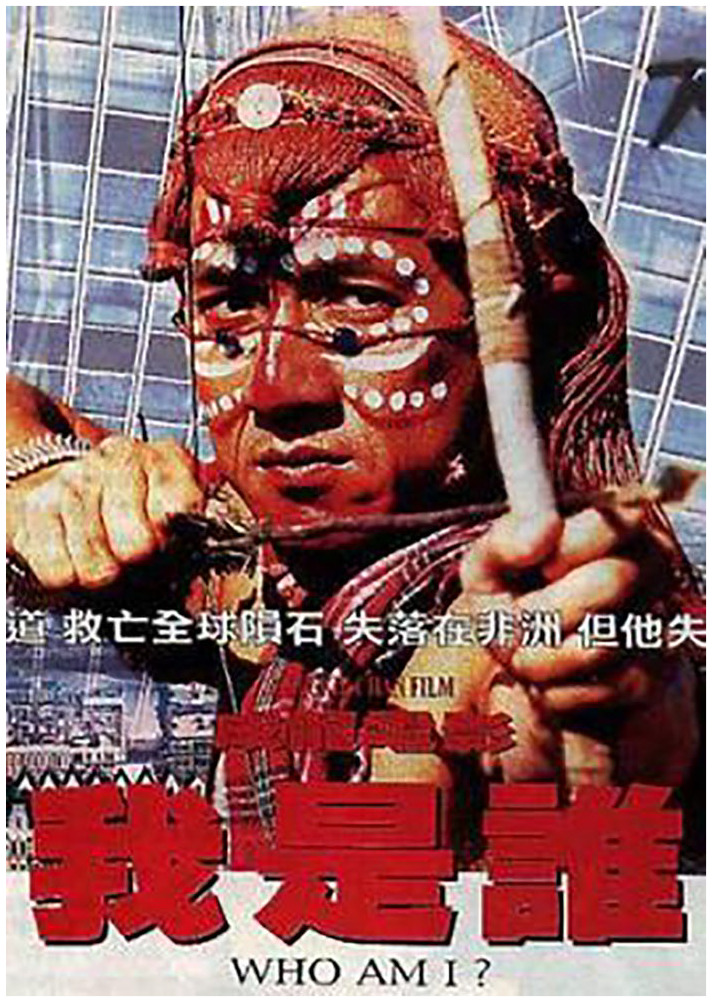
DVD cover from Jackie Chan’s leading role in the Kung Fu film *Who Am I* (1998).

**Figure 8.**
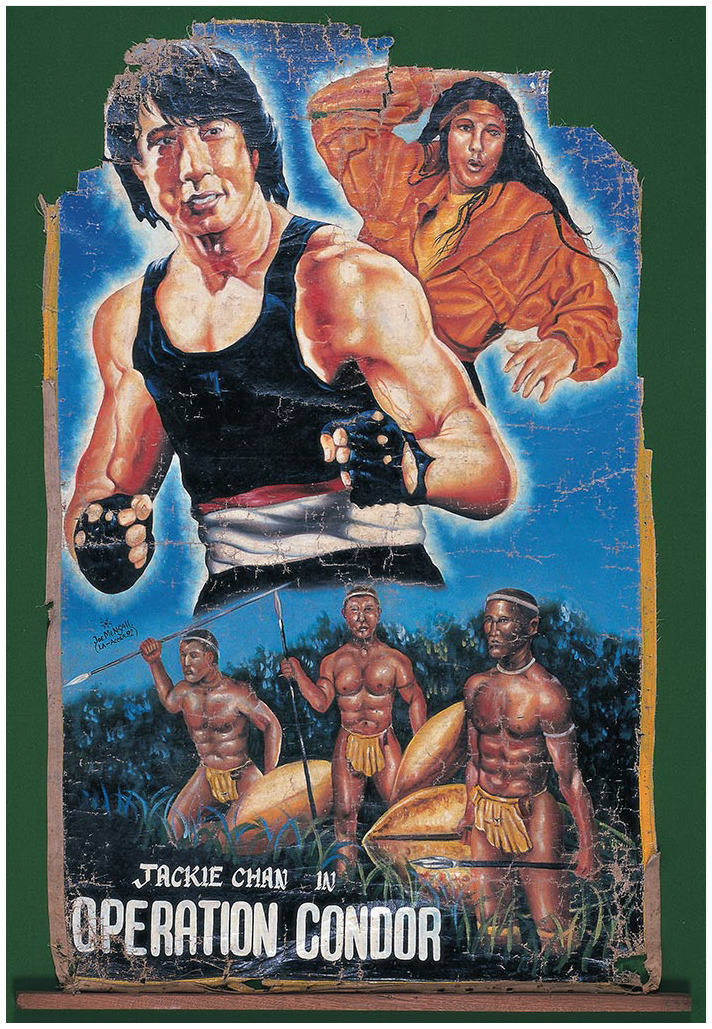
Film poster designed by Joe Mensah in Ghana for the movie *Operation Condor* (Jackie Chan, 1991). Oil on canvas. 64 × 40 inches. Source: Image courtesy of Ernie Wolfe Gallery.

Hand-painted movie posters reflect the appeal Chinese martial arts have for people’s imaginations in Africa. These posters reimagine movies to suit the artist’s vision (Figures 7 and 8). Africa sometimes plays a role in the narratives and plots of the Kung Fu films, as some of them have been shot in African locations. One favorite film among the Window of the World performers is *Who Am I?* (1998) starring Jackie Chan. After a botched Central Intelligence Agency (CIA) operation in South Africa, Chan’s character is rescued by a ‘tribe’ who take him under their wing. Suffering from amnesia, when asked his name, Chan responds by asking ‘Who am I?’ Several of the theme park performers cite this movie as having been instrumental in inspiring them to learn martial arts.

Writing about Kung Fu, and the role of martial arts generally in Africa, Rodrigue et al. (2019: 68) have highlighted that the cultural gaps leading to misinterpretation can also lead to new understandings. More specifically, the African reception of Chinese martial arts combined with African ontologies relating to bodily creative and spiritual practices like dance gives way to new conceptions and understandings of Kung Fu (Rodrigue et al., 2019: 76).

Like the virtual sculpture gardens floating in an ahistorical space, and the souvenir trinkets mass-produced in the manufacturing center of Shenzhen, output from the past is used as input for creating a backdrop for the present. In their depictions of African chiefs and kings, the performers at this park view themselves as avatars in a simulation of Africa. Avatars are stand-ins for deities; they are generally shape-shifting and surrounded by an aura. This is similar to how Thato describes his process of imitation, inspired by a Bruce Lee quote he memorized off the Internet: ‘Be formless, shapeless, like water. Now you put water into a cup, it becomes the cup. Put it into a teapot, it becomes the teapot. Now water can flow, or creep, or drip, or crash. Be like water my friend.’

Spivak’s (1996) concepts of strategic essentialism and self-essentializing practices are useful here, especially within the capitalist framework. By ‘being like water’, Thato and the other performers become shape-shifting actors in a marketable ethnic enterprise. It is both to earn a living in the present and to be able to plan for a more enterprising future. In the words of one performer:

One day I would like to put together my own ensemble of performers that I select myself. We would perform folkloric dances that are more faithful to what we showcase for tourists at home in South Africa. I think the Chinese public would like seeing these. But I am not sure the government will allow me to stay on without a formal contract with a big company like Window of the World.

This performer aspires to one day take charge of the stage shows, rather than continue to let Chinese art directors control the show. In addition to a national pride performed in and through cultural heritage, this performer also understands Chinese audiences to be deserving of a closer representation of different South African music and dance genres. Another performer said that he observes young Chinese people drawn to hip hop and, increasingly, Afro-dance. In his opinion, there is a growing market for this in cities all over China, and therefore a potential opportunity for him to earn a living outside the park.

Despite the role of the Chinese art director, performers view themselves as co-producers of this ‘Africa’ – a commodified spectacle consumed by the park’s customers, many of whom have never seen an African person before. These performers have achieved a level of mobility through an endorsement of their own exoticism, even as it is projected onto them by primordialist fantasies. The performers have a window of opportunity. However, many who have been working there for several years know that, for them, the possibilities for long-term future-building in China are limited.

## Coda

Window of the World, conceived in the 1990s for Chinese consumers, executes an agenda to promote new forms of leisure among the emerging middle class. It achieves this by controlling and commodifying local and global cultural scripts. The immersive experience promised by the park is partly fulfilled through live spectacles staged with real people. For many, travel in the physical sense continues to be an unattainable luxury, or something that they do not wish to experience with all of its inconveniences relating to radical cultural encounter. In its place, theme parks offer an opportunity to participate as consumers in a global tourist economy, albeit ‘global’ as defined by Chinese characteristics.

Since the 1990s, new migrations of people from Africa point to an intensification of Sino–African relations. Despite the western media’s negative spin on China’s neocolonial ambitions for the African continent, the African employees interviewed at the Window of the World theme park expressed ambivalence about the Sino–African relationship. Performers believe that working in China will contribute to increasing the breadth of their opportunities in the future. They understand that Africa–China state-level cooperation has facilitated this mobility. However, they are not ignorant of the asymmetries regarding how they, as Africans, are treated by the park management in contrast to other performers. Nonetheless, they continue to believe that working in China is a path toward a better future, even if they cannot currently articulate exactly how or why they believe this. Young people across the African continent are being scouted for their talent. Scouts promise jobs which lead to the physical and socio-economic mobility so desired by many young people in Africa. However, they must be willing to perform a vision of ‘Africa’ as envisioned and scripted by the park’s Chinese management. In this regard, many performers understand that they must ‘become like water’ to skirt around any struggle over some authentic portrayal of African ethnicity. The physical virtuosity exhibited by performers is itself informed by images which are essentialized as ‘the other’ and thrown into circulation. This results in a feedback loop, but one in which every revolution informs and modifies the past (and projected) cultural iterations which form its ever-circulating content.

The popularity of Kung Fu movies for many young people living in Africa has become a window of encounter with Asian culture, inspiring them to incorporate martial arts choreographic sequences into their own dance performances. Theme parks around the world have become sites of globalization discourses; they make visible the ways in which the global is expressed in the local. The Africa pavilion performers highlight how global circulations are first made local, then sent back out into the global again. Presented as a bounded cultural unit, the Africa pavilion is animated by young people who themselves have first encountered the idea of China through the cultural exports of movies. These performers inspire awe and wonder in Chinese audiences composed of individuals who may never have (nor want) the opportunity to travel to an African country.

The oft-cited analysis of the postmodern condition perhaps assumes new traction in the temporal context of COVID-19. These days, the virtual is entrenching itself in both our labor and leisure at an accelerated pace. With the onslaught of social distancing, lockdowns, travel bans and curfews, it is time for a consideration of the social, political and economic consequences of global travel; not to mention the ways in which global supply chains and global travel have the potential for spurring on local viral outbreaks into becoming global pandemics. Are we all destined to become post-tourists, having to satisfy ourselves with virtual access to the elsewhere? What form will cultural encounters take when they are mediated through platforms and screens? For the moment, we must gaze outside our own windows onto the world, one which is shaped by churning images that travel outside their own containers.

## Notes

1. Gerhard Hoffstaedter (2008: 145) discusses something similar in Malaysian theme parks.

2. For a theoretical discussion about kitsch as it relates to replication in the context of China, see Alberto Castelli (2021).
